# Aberrant Expression of Claudins in Head and Neck Carcinomas and Their Prognostic and Therapeutic Value: A Narrative Review

**DOI:** 10.3390/cancers15174208

**Published:** 2023-08-22

**Authors:** Tarek Ziad Arabi, Linah Abdulmohsen Algheryafi, Nora A. Alodah, Hamza M. Kossai Enabi, Amjad Abdullah Alshehry, Abderrahman Ouban

**Affiliations:** 1 College of Medicine, Alfaisal University, Riyadh 11533, Saudi Arabia; tarabi@alfaisal.edu (T.Z.A.); lalgheryafi@alfaisal.edu (L.A.A.); nalodah@alfaisal.edu (N.A.A.); henabi@alfaisal.edu (H.M.K.E.); aalshehry@alfaisal.edu (A.A.A.); 2 Department of Pathology, College of Medicine, Alfaisal University, Riyadh 11533, Saudi Arabia

**Keywords:** claudins, squamous cell carcinoma, head, neck, prognosis, expression, mechanisms

## Abstract

**Simple Summary:**

Head and neck cancer is one of the most common cancers worldwide and is associated with poor prognosis. Studies have demonstrated that claudins plays a significant role in the pathogenesis of cancers throughout the human body and highlights their prognostic role. The aberrant expression of various claudins has been strongly associated with carcinomas across the head and neck region. Additionally, the expression of these proteins predicts prognosis in several instances. Preclinical studies have highlighted that inhibiting the pathways which mediate altered claudins expression can halt neoplastic processes and minimize cellular invasion. Hence, understanding the role of claudins in head and neck carcinomas and their related mechanisms provides both prognostic and therapeutic value.

**Abstract:**

Head and neck carcinomas have been associated with poor prognosis. Recent studies have highlighted the role of claudins’ expression in tumors throughout the body, and their prognostic and therapeutic role. Understanding the role of claudins and how their expression affects the progression of carcinomas in the head and neck region may allow for advances in the prognosis and management of this type of cancer. Several studies have highlighted the aberrant expression of the proteins in carcinomas in this region. Specifically, the overexpression of claudin-1 and downregulation of claudins-4, -7, and -17 have been linked with poor survival in oral squamous cell carcinoma patients. In laryngeal squamous cell carcinoma, increased levels of claudins-1 and reduced levels of claudins-3, -8, and -11 have been linked with poor outcomes. Targeting these proteins has shown promising outcomes as therapeutic in preclinical studies. However, studies remain extremely limited in nasal and hypopharyngeal carcinomas. In this review, we survey the available literature describing the aberrant expression of various claudins in carcinomas in this region, while highlighting their potential prognostic and therapeutic value. Then, we describe some molecular mechanisms involved in the aberrant expression of claudins and how they can be utilized as therapeutic targets.

## 1. Introduction

The barrier function of the cells is performed by the epithelia, which forms a protective layer between the body’s interior milieu and the outside environment [1,2]. At the heart of this barrier function are the tight junction strands, composed of the occludin, zona-occludens, desmosomes, and claudins proteins. Tight junctions regulate the permeability of the epithelial barrier and limit passage through the paracellular space [3]. In addition to their barrier role in protecting the underlying structures, tight junctions help regulate cellular proliferation and differentiation [4,5,6,7]. Tight junctions will also ensure the maintenance of cellular polarity, a critical element in maintaining proper cellular communication and signaling [8,9,10].

Recent mounting evidence points to signaling pathways which converge on tight junction positions and interact with the proteins forming those tight junctions [10,11]. Claudin proteins have also been noted to regulate the cell cycle via the Zonula occludens 1-associated nucleic acid-binding protein, p27^kip1^, and SP1 [12]. The aberrant expression of these tight junction proteins may play critical roles in establishing alternative signaling and activation of neoplastic pathways, which converge upon the cell and result in its transformation [13]. This was further supported by several studies which have shown that in cancer, claudins exhibits changing levels, with some showing increased and others showing decreased expression levels [5,14,15,16,17]. More evidence was presented with the manipulation of claudins levels in in vitro and animal studies, which resulted in the regression of aggressive tumor traits, especially cell motility, invasion, and metastases [5,14,15]. Additionally, claudin-6 has been shown to promote collective cell migration to the leading front of neoplastic cell lines [18]. In addition to their roles as tight junction proteins, the role of claudins in neoplastic development includes aberrant signaling [19,20,21], modulating autophagy [20,22,23,24], mediating the epithelial–mesenchymal transition phenomenon [19,25,26,27], mediating bacterial and viral oncogenesis [27,28,29], mediating the stemness of cancer cells [30], and the maintenance of the tumor microenvironment [30,31]. The above is added to the long-established role played by claudins’ knockdown in the development of cancer, allowing for the possible passage of toxins and carcinogens into the deeper layers of the epithelium. Collectively, the above evidence of claudins’ postulated roles in cancer development and progression suggests a great potential for these proteins in the fight against cancer and its complications.

Head and neck squamous cell carcinoma is the sixth most common tumor worldwide [32]. Neoplasms in this region present several challenges including old age at presentation, resulting in impaired functional status and comorbid medical conditions [33]; advanced stage at presentation [34]; significant toxicity associated with multimodality therapy [35]; multifocal disease; and the associated difficulty in mapping it, resulting in an increased likelihood of residual disease and recurrence [35]. Head and neck cancers are a challenging group of neoplasms which require additional strategies to counter, including the targets of molecular-era treatments.

Emerging evidence has highlighted the presence of irregular claudins expression in head and neck carcinomas. For example, Ouban et al. demonstrated that claudin-1 is overexpressed in carcinomas of several organs in the head and neck regions [17]. Additionally, overexpression of the same protein has been strongly associated with lymphatic invasion and poor overall survival in head and neck carcinoma patients [36]. Hence, studies have described the associations between head and neck carcinomas and claudins expression. Furthermore, studies have demonstrated that the level of expression of various claudins provides significant prognostic and clinicopathological value. Understanding the mechanisms underlying abnormal claudins expression in head and neck carcinomas may also provide the basis for therapeutic targets in future trials. This paper will provide a comprehensive review of the dysregulated expression of claudins in head and neck cancer and its possible utilization in the management of head and neck cancers. Then, we describe some of the molecular mechanisms resulting in irregular claudins expression and how they can be targeted in the management of such tumors.

## 2. Search Strategy

A literature search was conducted on the PubMed database using the following keywords: head and neck, mouth, tongue, lip, tonsil, salivary gland, larynx, nose, hypopharynx, nasopharynx, and carcinoma. Additionally, the references cited by the retrieved publications were manually reviewed. Irrelevant studies were excluded, and a narrative review was drafted accordingly.

## 3. Claudins Expression in the Normal Head and Neck Regions

The expression of claudins varies greatly between the different organs in the head and neck regions. For example, claudin-1 stains strongly in the junctional epithelium of the gingiva of rats [37]. Claudin-1 expression has been reported to be low in both tongue and palate squamous epithelium [17,38]. In salivary glands, claudin-1 can be seen in both acinar glands and ductal epithelial cells [17]. On the other hand, claudin-3 staining is strong in the granular and spinous layers of the gingival epithelium of rats [37]. Studies have also documented an intense expression of claudin-4 in the stratum germinativum and stratum spinosum, and a moderate expression in the stratum planocellulare in tissues from the hypopharynx, oropharynx, and larynx, while claudins-3, -8, and -10 are negative in the normal epithelium of these sites [39]. Understanding the expression of claudins proteins in normal epithelium across various regions of the head and neck is crucial to highlighting the role of aberrant claudins expressions.

## 4. Claudins Expression in Oral Squamous Cell Carcinoma

Although the exact role of claudins is not very well understood in cancer progression, claudin-1 is a frequently studied protein in the literature. In oral squamous cell carcinoma (OSCC), claudin-1 expression levels ranged from high to no change [40]. Over-expression of claudin-1 correlates with higher histological grades, specifically when the protein is positioned intracellularly, compared to when it is membranous or cytoplasmically expressed [37]. Although several studies have found changes in claudin-1 expression in OSCC, its prognostic value appears to be limited (Table 1). One study by Upadhaya et al. found reduced survival rates among patients with increased claudin-1 levels; however, these findings were not statistically significant [37]. Another study reported that claudin-1 upregulates the activity of matrix metalloproteinases (MMP_, which in turn increases OSCC invasiveness [41]. The same study also found no linkage between claudin-1 expression and patient health outcome. Zejc et al. reported similar results, where claudin-1 expression was not correlated to tumor stage, expansion, histological differentiation, recurrence-free survival, and several other clinicopathological parameters [40]. On the other hand, reports have found significant positive associations between pathological grade, perineural and vascular invasion, nodal metastasis, and advanced tumor staging [42]. Monteiro et al. also found that the combined usage of claudin-1 and occludin expression levels independently predicts recurrence-free and cancer-specific survival in OSCC patients [43]. Additionally, it has been reported that concurrent detection of elevated claudin-1 and junctional adhesion molecule-A (JAM-A) levels is associated with poor survival rates in this patient population [37]. Hence, the prognostic utility of claudin-1 may be improved by combining it with the expression of other adhesion molecules.

Claudin-2 is significantly upregulated in OSCC, with evidence that overexpression is independently linked to poor recurrence-free survival, tumor stage, and lymphatics involvement [37]. However, it is worth noting that another study found that increased claudin-2 expression is associated with improved survival in patients with squamous cell carcinomas in the head and neck regions [39]. Therefore, future studies should focus on understanding the true prognostic value of claudin-2 in OSCC. Claudin-3, on the other hand, is not expressed in cancerous or normal epithelium [39].

In OSCC tissue, claudins-4, -5 and -7 are expressed significantly lower than normal tissue [39,40]. Specifically, downregulation of claudin-4 has been reported to predict reduced recurrence-free survival [40]. Positive expression of the same protein has been linked to decreased perineural infiltration [53], while other studies have found no correlation between its expression and clinicopathological parameters [42]. Claudin-7 expression has been found to be a good indicator for the prognosis and recurrence rate in OSCC patients [40]. The knock-down of this protein is associated with higher pathologic grade, advanced staging, increased perineural, vascular and lymphatic invasions, and reduced survival [40,56]. Li et al. studied the effects of the interferon regulatory factor-2 (IRF2) on claudin-17 expression in human OSCC cell lines [57]. IRF2-induced claudin-7 transcription resulted in reduced proliferation, invasion, and migration of the cell lines [57]. These findings may demonstrate the possible utility of IRF2 as a therapeutic target in the future. Lastly, claudin-17 gene expression has been shown to decrease in OSCC and reduced levels have been associated with poor TNM staging and survival [58]. Collectively, claudins-1, -2, -4, -7, and -17 have proven valuable in the prognosis of OSCC patients (Table 1 and Table 2).

## 5. Claudins Expression in Tongue Squamous Cell Carcinoma

Only a few claudins have been discussed with regards to their involvement in tongue squamous cell carcinoma (TSCC) (Table 1 and Table 2). Bello et al. analyzed the expression of claudins-1, -4, -5, and -7 in TSCC, where they observed a strong expression for claudins-1 and -7, medium immunoreactivity for claudin-4, and low immunoreactivity for claudin-5 [44]. The number of cells stained in the superficial parts of the tumors with claudins-4 and -7 correlated with the tumor stage. A moderate immunohistochemical score with claudin-7 in TSCC was associated with better survival. When they compared between the superficial versus the invasive fronts of the tumors, high staining intensity of claudin-7 in the invasive front of the tumors was associated with decreased patient survival. It has been documented in a study of 83 TSCC specimens that high cytoplasmic expression of claudin-1 is linked to cervical lymph node metastasis, but not with disease progression [45]. The study suggested that the ectopic, intracellular location of claudin-1 at the invasive front may be an additional and promising diagnostic marker of TSCC [45]. Although claudin-7 has shown promising results as a prognostic tool in TSCC, further studies are needed to confirm its role and the role of other claudins in the neoplasm.

## 6. Claudins Expression in Lip Squamous Cell Carcinoma

Studies reporting claudins expression in lower lip squamous cell carcinomas (LLSCC) are limited (Table 1). De Aquino et al. found that claudin-1 exhibited a stronger expression in metastatic LLSCC than in nonmetastatic tumors and a higher expression in advanced stages of LLSCC (stages III and IV) than in the lower stages (I and II) [51]. To the best of our knowledge, this is the only available study analyzing claudins expression in LLSCC.

## 7. Claudins Expression in Tonsillar Squamous Cell Carcinoma

Among the claudins, only claudins-1 and -7 have been identified in tonsillar squamous cell carcinoma, with claudin-1 being highly expressed and claudin-7 exhibiting low levels of expression in all stages of cancer development [46] (Table 1 and Table 2). These changes were seen as independent of human papilloma virus infection.

## 8. Claudins Expression in Salivary Gland Carcinomas

The relationship between claudins expression and the clinical–pathological parameters has been assessed in salivary gland carcinomas. Arruda et al. examined the effect of epidermal growth factor (EGF) on the gene expression of claudins in salivary gland carcinomas [47]. Most mucoepidermoid carcinomas showed highly expressed claudins-1, -3, -4, -5, and -7 (Table 1). Patients older than 40 and Caucasians had higher levels of claudin-1 expression. In vitro tests showed a propensity for claudins gene expression to rise in response to EGF stimuli. In line with these findings, concurrent treatment with EGF and vascular endothelial growth factor antagonists has been shown to inhibit neoplastic growth and metastasis in mice models with adenoid cystic carcinoma [63]. The biological understanding of these proteins’ expression and associated signaling pathways will serve as a foundation for future research into potential target therapies.

In a second study, Abd El-Ghani et al. assessed the claudin-4 gene and protein expression via real-time PCR, along with immunohistochemistry in 30 specimens containing normal salivary tissue, pleomorphic adenomas, Warthin tumors, mucoepidermoid carcinomas, and adenoid carcinomas [60]. Their study revealed that claudin-4 is strongly expressed in normal salivary glands and benign salivary tumors. On the contrary, malignant cells’ claudin-4 protein expression levels were moderate to weak [45], contrary to the study by Arruda et al. [47]. Therefore, further studies are needed to confirm the levels of expression of claudin-4 in salivary gland carcinomas.

A third study reported claudin-3 modulation via the tumor necrosis factor (TNF)-α through extracellular signal-regulated kinase (ERK) 1/2/slug signaling in the submandibular gland [64]. The TNF-α-induced downregulation of claudin-3 was identified in the submandibular gland, whereas the signaling pathway had no effect on claudins-1 and -4. Claudin-4 levels are also significantly increased in the irradiated submandibular glands of rat models [65]. Radiation-induced alterations, however, can be attenuated via pilocarpine administration, which heals the damage to tight junctions and restores claudin-4 levels [65].

A study investigating claudin-7 expressions in salivary adenoid cystic carcinomas revealed decreased claudin-7 expression via both immunohistochemistry and Western blot [61]. The reduced expression of the protein correlated well with lymph node metastases, recurrence, and gender. Through the analysis of a stably transfected claudin-7 knockdown of an adenoid cystic carcinoma cell line and a subcutaneous tumor formation model, it was evident that the tumorgenicity of the aforementioned cell lines was due to regulation of an epithelial–mesenchymal transition via the Wnt/β-catenin signaling pathway. Claudins in salivary gland carcinomas have been largely studied, leading to the discovery of signaling pathways to be used as potential therapeutic targets.

## 9. Claudins Expression in Laryngeal Squamous Cell Carcinoma

The expression of several claudins has been studied in laryngeal squamous cell carcinomas (LSCC) (Table 1 and Table 2). The mRNA and protein expression of claudins-3 and -8 has been found to be overexpressed in LSCC, while claudins-1 and -7 are significantly downregulated [52]. Similar results can also be seen using immunohistochemistry [52]. Upregulation of claudin-3 has been associated with distant metastasis, while claudin-8 is associated with histological grade and Ki-67 expression [52]. On the other hand, downregulation of claudin-7 correlates with distant metastasis [52]. Moreover, analyses have been conducted on the relationships between claudins expression and LSCC patient survival. Expression of claudins-1 and -7 is significantly associated with improved survival times, while the opposite can be seen in regards to claudins-3 and -8 expression [52]. It is worth noting, however, that reports of claudin-7 in LSCC are conflicting. Kapral et al. found no significant difference between the LSCC and normal specimens in regard to claudin-7’s transcriptional activity [66]. Hence, it is important to interpret the results of claudin-7 in LSCC with caution until further reports are revealed.

Claudin-4’s role in LSCC remains unclear. Liu et al. demonstrated that the expression of claudin-4 is downregulated in LSCC and its expression is negatively correlated with the methyl-CpG-binding protein 2 (MeCP2), a prototype transcriptional repressor [59]. Additionally, neoplastic cell migration and invasion of LSCC cell lines are inhibited when claudin-4 DNA is demethylated via 5-aza-2′-deoxycytidine (a DNA methyltransferase inhibitor), but are recovered when its DNA is silenced [59]. The study by Liu et al. demonstrates that DNA methylation, likely through MeCP2 expression, is key for claudin-4 downregulation and tumor expansion. On the contrary, Xu et al. studied the effects of circular RNA SERPINA3 (circSERPINA3) on LSCC invasion and migration [54]. First, the authors found that circSERPINA3 is significantly upregulated in LSCC cell lines and tissues and downregulates microRNA (miR)-855-5p expression. Interestingly, the study then demonstrated that the silencing of circSERPINA3 and overexpressing of miR-855-5p suppresses the epithelial–mesenchymal transition by inhibiting claudin-4, snail, and vimentin expression and increasing E-cadherin expression [54]. Hence, further studies are needed to clarify whether claudin-4 plays a pro-oncogenic or anti-oncogenic role in LSCC.

Lastly, Shen et al. studied the clinical significance of claudin-11 promoter hypermethylation in LSCC using a quantitative methylation-specific polymerase chain reaction [62]. They reported that claudin-11 promoter methylation increased in neoplastic LSCC tissues compared to adjacent non-neoplastic tissues. The authors also found that increases in claudin-11 promoter methylation are associated with lymph node metastasis, advanced clinical stages, increased T classifications, and poor overall survival [62]. As a result, claudin-11 methylation plays a major role in the neoplastic processes of LSCC.

## 10. Claudins Expression in Nasal Squamous Cell Carcinoma

To the best of our knowledge, studies have yet to establish a relationship between nasal squamous cell carcinoma and claudins expression. Studies are urgently needed to identify the prognostic and therapeutic value of claudins in the neoplasm, as the 5-year overall survival rate is 50% [67].

## 11. Claudins Expression in Hypopharyngeal Squamous Cell Carcinoma

Immunohistochemical studies have demonstrated that claudin-1 is significantly upregulated in hypopharyngeal squamous cell carcinoma (HSCC) compared to non-neoplastic cells [49,50] (Table 1). Additionally, claudin-1 overexpression is significantly correlated with poor differentiation, lymph node metastasis, and survival rates among HSCC patients [49,50]. It has been hypothesized that claudin-1 induces tumor lymphatic vessel generation, thereby enhancing the spread of the tumor [50]. To the best of the authors’ knowledge, the role of other claudins proteins in HSCC has not yet been investigated.

## 12. Claudins Expression in Nasopharyngeal Squamous Cell Carcinoma

In a study by Hsueh et al., an increased expression of claudins-1 and -4 and a low expression of claudin-7 were seen via immunohistochemistry in nasopharyngeal carcinoma (NPC) [48] (Table 1 and Table 2). Low claudin-4 expression and high claudin-7 expression were correlated with distant metastases. Increased claudin-7 was also correlated with high tumor stages. Furthermore, decreased claudin-4 and increased claudin-7 expressions were independent risk factors for reduced distant metastases-free survival among these patients [48]. In a second study by Lee et al. assessing the role of claudin-1 in NPC, the authors reported an increased expression of claudin-1 in NPC cell lines when treated by serum-deprivation or with fluorouracil [68]. Increases in claudin-1 were associated with significant anti-apoptotic effects. Restoring E-cadherin significantly reduced claudin-1 levels and reversed the associated anti-apoptotic effects of the protein, suggesting a negative regulatory role of E-cadherin on claudin-1 in NPC.

Kojima et al. further assessed the expression of claudins in Epstein–Barr virus-associated non-keratinizing NPC [55]. The authors reaffirmed the overexpression of claudin-1 seen in NPC. The authors also demonstrated that claudin-4 is expressed in all specimens, claudin-2 was not expressed in any, and claudin-3 showed a variable expression among the samples. Given the fact that claudins-3 and 4 are receptors for cytotoxic *Clostridium perfringens* enterotoxin (CPE), CPE has drawn attention for malignant tumors expressing the two proteins [55]. CPE damages cells through osmotic disruption, leading to cell lysis [69]. The enterotoxin has shown promising results in claudins, inducing rapid cytolysis in claudin-3 and claudin-4-positive breast cancer cells [69]. Studies are needed to determine whether such effects can be reproduced in NPCs.

## 13. Mechanisms of Aberrant Claudins Expressions in Head and Neck Cancers and Their Therapeutic Value

**Figure 1:**
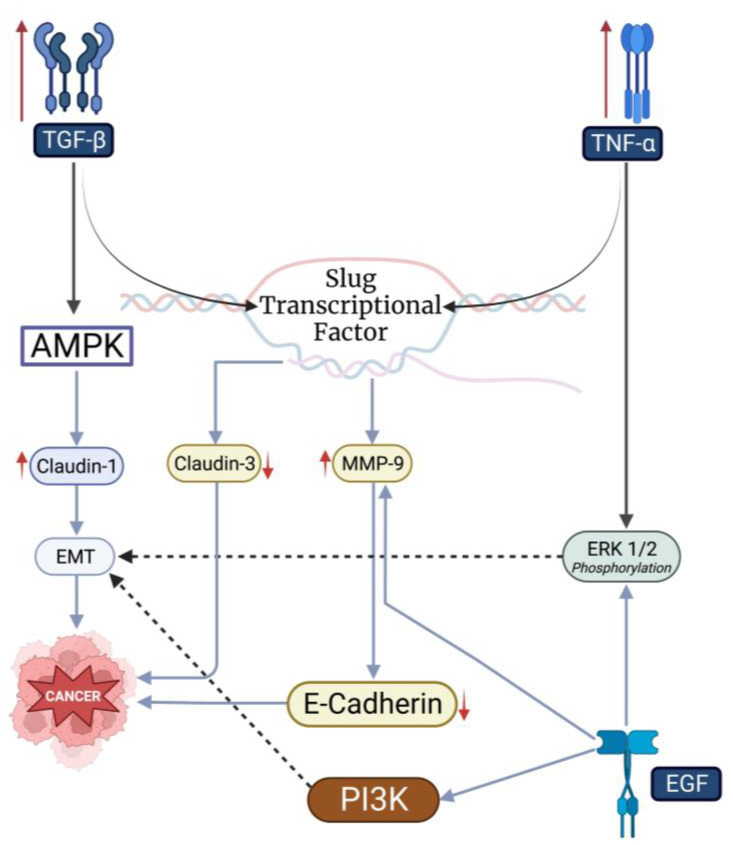
TGF-β, TNF-α, and EGF play a crucial role in neoplastic processes through the modulation of claudins expression in head and neck carcinomas. This figure was generated using biorender.com accessed on July 31 2023. ↑: increased activity; ↓: decreased activity.

The mechanisms underlying the aberrant expression of claudins in head and neck carcinomas are unclear. Although several studies have highlighted such processes in other cancers, studies remain limited in the head and neck regions in this regard. Chang et al. found that claudin-1 overexpression was negatively associated with AMP-activated protein kinase (AMPK) and is positively associated with the transforming growth factor (TGF)-β (Figure 1) [70]. Accordingly, AMPK activation overrides claudin-1-induced tumor invasion, suggesting a role of a claudin-1/AMPK/TGF-β axis in these tumors [70]. Indeed, it has been previously demonstrated that TGF-β promotes epithelial–mesenchymal transition and suppresses anti-neoplastic immune responses [71], and patients with elevated TGF-β mRNA levels have poor overall survival in silico [72]. Additionally, vactosertib, a TGF-β receptor inhibitor, exhibits significant antineoplastic effects on head and neck cell lines [72]. Clinically, bintrafusp alfa, a bifunctional function protein targeting TGF-β and programmed death-ligand-1, has shown clinical efficacy and a safe profile in patients with advanced head and neck squamous cell carcinomas in a phase I cohort study [73]. Hence, targeting the claudin-1/AMPK/TGF-β may prove as a plausible therapeutic target for head and neck cancer patients overexpressing claudin-1 and TGF-β.

As previously stated, TNF-α plays a key role in claudin-3 downregulation in submandibular gland neoplasms (Figure 1) [64]. TNF-α increases ERK1/2 phosphorylation and transcriptional factor slug expression [64]. Slug knockdown reduces claudin-3 expression in the submandibular glands [64]. TGF-β has been shown to result in slug-mediated increases in MMP-9 in OSCC cells, thereby enhancing tumor invasion [74]. Additionally, overexpression of the transcriptional factor correlates with poor disease recurrence and disease-free survival in LSCC patients and chemo- and radiotherapy resistance [75,76]. Interestingly, head and neck carcinoma patients with slug overexpression have a 3.3-times better chance of survival with upfront surgery compared to primary chemo- or radiotherapy [76]. Collectively, these results demonstrate that the slug transcriptional factor plays a critical role in the pathogenesis of head and neck carcinomas.

EGF and its accompanying receptor have also been a highlight of studies on head and neck carcinomas (Figure 1) [77]. EGF promotes aberrant claudins expression and neoplastic invasion [47]. EGF has been shown to regulate claudin-2 and -4 expressions through Src and STAT3 pathways in kidney cells [78]. Accordingly, the targeted inhibition of STAT3 in tumor-associated myeloid cells sensitizes squamous cell carcinomas to radiotherapy and T-cell-mediated immunity in human-to-mice xenotransplantation models [79]. In head and neck squamous cell carcinoma cell lines, treatment with EGF promotes cell invasion via ERK1/2- and PI3K-mediated epithelial–mesenchymal transitions and E-cadherin degradation via MMP-9 [80]. Overall, targeting TGF-β, TNF-α, and EGF pathways has shown promising results in preclinical studies. Clinical studies are needed, however, to determine whether these findings can be translated to patients.

## 14. Conclusions

Carcinomas of the head and neck remain a therapeutic challenge to this day, as a result of late age at presentation and rapid spread of the tumor. Recently, the role of claudin proteins in the development and progression of the neoplastic has fallen under the spotlight of the scientific community across various tumors of the human body. Studies have highlighted the aberrant expression of proteins that are extensively in the head and neck regions. Specifically, overexpression of claudin-1 (in combination with other adhesion molecules) and downregulation of claudins-4, -7, and -17 have been linked with poor survival in OSCC patients. In LSCC, increased levels of claudin-1 and reduced levels of claudins-3, -8, and -11 have been linked with poor outcomes.

We also summarize key molecular mechanisms regulating claudins expression in head and neck squamous cell carcinomas. The claudin-1/AMPK/TGF-β axis appears to be a key mediator of the epithelial–mesenchymal transition and neoplastic invasion. Both preclinical and clinical studies have demonstrated positive results. Additionally, TNF-α/slug pathways promote neoplastic processes by enhancing the epithelial–mesenchymal transition and increasing MMP-9 release. Lastly, EGF has been shown to modulate claudins expression via Src and STAT3 pathways and promote TNF-α-like effects through ERK1/2 and PI3K signaling.

However, our manuscript also highlights a lack of studies in some types of squamous cell carcinoma, especially nasal carcinomas and HSCC. Therefore, future studies are needed to demonstrate the expression of claudins and their role in the pathogenesis of these neoplasms. Emerging evidence has also identified several signaling pathways involving claudins with promising therapeutic results. Clinical studies are needed to confirm whether these outcomes reflect true clinical settings.

## Figures and Tables

**Table 1:** Claudins showing overexpression in various carcinomas in the head and neck regions. (* conflicting data).

Claudins Overexpression	Type of Cancer	Clinical Significance	Methodology	Reference(s)
1	OSCC	Poor recurrence-free and cancer-specific survival *	IHC and Western blot	[37,40,41,42,43]
	TSCC	Increased cervical lymph node metastasis	IHC	[44,45]
	Tonsillar squamous cell carcinoma	N/A	IHC and PCR	[46]
	Mucoepidermoid carcinomas	N/A	IHC and PCR	[47]
	NPC	N/A	IHC	[48]
	HSCC	Poor differentiation, lymph node metastasis, and reduced survival rates	IHC	[49,50]
	LLSCC	Advanced tumor staging	IHC	[51]
2	OSCC	Shorter recurrence-free survival *	IHC	[37,39]
3	Mucoepidermoid carcinomas	N/A	IHC and PCR	[47]
	LSCC	Distant metastasis and poor	PCR and Western blot	[52]
4	OSCC	Increased perineural infiltration and poor recurrence-free survival	IHC	[39,41,53]
	Mucoepidermoid carcinomas *	N/A	IHC and PCR	[47]
	LSCC *	N/A	Western blot	[54]
	NPC	N/A	IHC	[48,55]
5	Mucoepidermoid carcinomas	N/A	IHC and PCR	[46]
7	TSCC	Poor survival	IHC and Western blot	[44]
	Mucoepidermoid carcinomas	N/A	IHC and PCR	[47]
	NPC	Distant metastasis, increased tumor stages, and poor distant metastases-free survival	IHC	[48]
8	LSCC	Poor histological grade and survival	PCR and Western blot	[52]

OSCC: oral squamous cell carcinoma; TSCC: tongue squamous cell carcinoma; NPC: nasopharyngeal carcinoma; HSCC: hypopharyngeal squamous cell carcinoma; LLSCC: lower lip squamous cell carcinoma; LSCC: laryngeal squamous cell carcinoma; IHC: immunohistochemistry; PCR: polymerase chain reaction; N/A: not available.

**Table 2:** Claudins showing under-expression in various carcinomas of the head and neck. (* conflicting data).

Claudins Underexpression	Type of Cancer	Clinical Significance	Methodology	Reference(s)
1	LSCC	Poor survival	IHC	[52]
2	NPC	N/A	IHC	[55]
4	OSCC	Poor recurrence-free survival and increased perineural infiltration *	Western blot and IHC	[40,41,42,53]
	NPC	Poor distant metastases-free surivval	IHC	[48]
	LSCC *	N/A	IHC and PCR	[59]
	Salivary gland carcinomas *	N/A	IHC and PCR	[60]
5	OSCC	N/A	IHC and Western blot	[39,40]
	TSCC	N/A	IHC	[44]
7	OSCC	High pathological grade, advanced staging, increased perineural, vascular, and lymphatic invasions, and poor survival	Western blot and IHC	[40,56]
	Salivary adenoid cystic carcinoma	Increased lymph node metastasis and recurrence	IHC, PCR, Western blot, and IFC	[61]
	Tonsillar squamous cell carcinoma	N/A	IHC and PCR	[46]
	LSCC *	Poor survival and distant metastasis	PCR and Western blot	[52]
11	LSCC	Lymph node metastasis, advanced clinical stages, increased T classifications, and poor survival (correlates with claudin-11 promoter hypermethylation)	PCR	[62]
17	OSCC	Poor TNM staging and survival	PCR, IHC, and Western blot	[58]

LSCC: laryngeal squamous cell carcinoma; NPC: nasopharyngeal carcinoma; OSCC: oral squamous cell carcinoma; TSCC: tongue squamous cell carcinoma; IHC: immunohistochemistry; PCR: polymerase chain reaction; IFC: immunofluorescence; N/A: not available.

## Data Availability

No data were generated in the drafting of this manuscript, and all original data may be found in the referenced articles.
